# Gastric Pneumatosis: An Atypical Presentation of Desmoid Tumor

**DOI:** 10.5811/cpcem.2021.5.50803

**Published:** 2021-08-27

**Authors:** Catarina Jorge, Miguel Varela, Júlio Ricardo Soares, Hugo Uribe, Luis Flores, Javier Moreno

**Affiliations:** * University Hospital Centre of Algarve - Faro Unit, Department of Internal Medicine, Faro, Portugal; † University Hospital Centre of Algarve - Faro Unit, Department of Emergency and Intensive Care, Faro, Portugal; ‡ University Hospital Centre of Algarve - Faro Unit, Department of Anesthesiology, Faro, Portugal

**Keywords:** Gastric pneumatosis, desmoid tumor, septic shock

## Abstract

**Case presentation:**

A middle-aged woman presented to the emergency department with a chief complaint of abdominal pain, fever, vomiting, and diarrhea. Abdominal computed tomography revealed gastric pneumatosis and air in the portal system. The patient had an unfavorable clinical course with pneumoperitoneum and septic shock due to secondary peritonitis. She underwent emergency laparotomy where neoformation of the mesentery root was found with infiltration of the small intestine and jejunal perforation. The anatomopathological study of the tumor revealed that it was a desmoid tumor.

**Discussion:**

To our knowledge this is the first report in the literature of gastric pneumatosis as the initial presentation of a mesenteric desmoid tumor. Although it usually has a benign clinical course, its locally invasive characteristics can lead to critical illness. Physicians shouldn’t overlook these types of complications, as early identification and surgical intervention can modify the prognosis and shorten hospital stay.

## CASE PRESENTATION

A 52-year-old woman with no pertinent past medical history presented to the emergency department for vomiting and diarrhea for one week. The patient also experienced fever and abdominal pain over the previous 24 hours. Upon initial evaluation, she was alert, hemodynamically stable, and afebrile. The abdomen was soft and depressible, nontender and nondistended. Laboratory tests showed an increased lactic acid of 2.5 millimoles per liter (mmol/L) (reference range**:** 0.5–2 mmol/L), and C-reactive protein of 149 milligrams (mg)/L (<5 mg/L), unchanged hepatic and renal function tests, without other relevant changes. Abdominal-pelvic computed tomography (APCT) revealed marked gastric distension with gastric pneumatosis (GP), parietal thickening of the jejunoileal loops, and air in the portal system (Image).

Upper gastrointestinal endoscopy revealed no disruption of the gastric mucosa or signs of ischemia. Exploratory laparoscopy was performed showing no evidence of visceral pathology. Subsequently, due to the worsening of abdominal pain and fever, a new APCT was ordered, revealing pneumoperitoneum and peritoneal fluid. Emergent laparotomy was performed, which revealed jejunum perforation with peritonitis. Exploration of the abdominal cavity found neo-formation of the root of the mesentery with infiltration of the small intestine. The patient had a segmental enterectomy and excision of tumor of the mesentery (7 centimeters), which was adherent to the intima of the third portion of the duodenum, the jejunum, and invasion of the superior mesenteric artery.

After operative repair, the patient was admitted to the intensive care unit (ICU) due to septic shock most likely caused by peritonitis. She had a prolonged hospitalization in the ICU for a total of 24 days, with several surgical revisions, requiring invasive ventilation for 19 days and vasopressor support for five days. Her condition gradually improved, due to antibiotics and surgical revisions, with resolution of organ dysfunctions. Histological analysis of neoformation revealed that it was an intra-abdominal desmoid tumor (DT).

The patient was discharged from the hospital after 45 days and was followed up with oncology consultation. After six months of follow-up, she presented with tumor recurrence and, in discussion with a solid tumors reference center, it was decided to start systemic treatment with tamoxifen and indomethacin.

## DISCUSSION

Gastric pneumatosis is a rare radiological entity defined by the presence of air inside the gastric wall.^1^ Pneumatosis intestinalis can occur in any part of the gastrointestinal tract, although GP is an uncommon localization.^2^ Gastric pneumatosis can be caused by ischemia, infection, mucosal disruption, endoscopic procedure, connective tissue diseases and, rarely, by tumors.^1^–^3^ Desmoid tumors are benign but locally invasive and rare, with an estimated prevalence of 0.03%.^4^ Only one case of abdominal DT with hepatic pneumatosis has been reported.^5^

The present case represents the first, atypical, presentation of a DT, which usually has a benign clinical course, but its locally invasive characteristics can lead to critical illness, such as this one. Early identification and urgent surgical intervention influence the prognosis. This image should raise awareness for a possible neoplastic entity as a differential diagnosis, not forgetting that complications may occur as well as the possible need for intensive care.

**CPC-EM Capsule**

What do we already know about this clinical entity?

*Gastric pneumatosis (GP) is a rare radiological entity defined by the presence of air inside the gastric wall. Desmoid tumor (DT) is unusual and benign, but locally invasive and can cause critical illness.*

What is the major impact of the image(s)?

*An atypical case of GP as the first manifestation of a mesenteric DT, which is properly illustrated in this image, but never before reported in the literature.*

How might this improve emergency medicine practice?

*Raising awareness for neoplastic etiology of GP and its possible complications that may require intensive care. Its early identification and urgent surgery modify the prognosis.*

## Figures and Tables

**Image:**
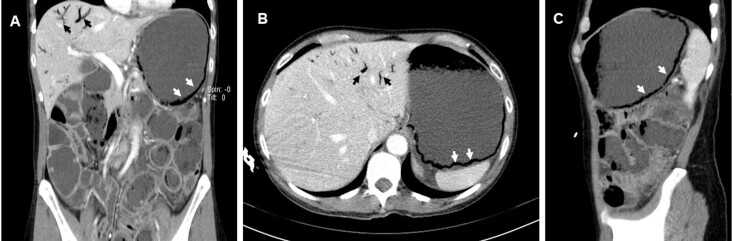
Abdominal-pelvic computed tomography (A - coronal; B- axial; C- sagittal) showing gastric pneumatosis (white arrow) and hepatic portal venous gas (black arrow).
